# Therapeutic approach to Class II, Division 1 malocclusion with maxillary functional orthopedics

**DOI:** 10.1590/2176-9451.20.4.099-125.sar

**Published:** 2015

**Authors:** Aristeu Corrêa de Bittencourt, Armando Yukio Saga, Ariel Adriano Reyes Pacheco, Orlando Tanaka

**Affiliations:** 1 MSc in Dentistry, Orthodontics, Uningá, Maringá, Paraná, Brazil; 2 Professor at the Specialization course in Orthodontics, Pontifícia Universidade Católica do Paraná (PUCPR) and ABO-PR, Curitiba, Paraná, Brazil; 3 PhD resident in Dentistry, Orthodontics, Pontifícia Universidade Católica do Paraná (PUCPR), Curitiba, Paraná, Brazil; 4 Full professor of Dentistry, Orthodontics, Pontifícia Universidade Católica do Paraná (PUCPR), School of Health and Biosicences, Curitiba, Paraná, Brazil

**Keywords:** Interceptive orthodontics, Class II, Klammt, Activator

## Abstract

**INTRODUCTION:**

Interceptive treatment of Class II, Division 1 malocclusion is a challenge orthodontists commonly face due to the different growth patterns they come across and the different treatment strategies they have available.

**OBJECTIVE:**

To report five cases of interceptive orthodontics performed with the aid of Klammt's elastic open activator (KEOA) to treat Class II, Division 1 malocclusion.

**METHODS:**

Treatment comprehends one or two phases; and the use of functional orthopedic appliances, whenever properly recommended, is able to minimize dentoskeletal discrepancies with consequent improvement in facial esthetics during the first stage of mixed dentition. The triad of diagnosis, correct appliance manufacture and patient's compliance is imperative to allow KEOA to contribute to Class II malocclusion treatment.

**RESULTS:**

Cases reported herein showed significant improvement in skeletal, dental and profile aspects, as evinced by cephalometric analysis and clinical photographs taken before, during and after interceptive orthodontics.

## Abstract (pt)

**INTRODUÇÃO:**

o tratamento interceptor da má oclusão de Classe II primeira divisão é um desafio comum para os ortodontistas, em função dos diferentes padrões de crescimento que podem ser encontrados e das estratégias de tratamento disponíveis.

**OBJETIVO:**

apresentar cinco casos de interceptação da má oclusão de Classe II primeira divisão usando o ativador aberto elástico de Klammt (AAEK).

**MÉTODOS:**

o tratamento dessa má oclusão pode ser realizado em uma ou duas fases; e a utilização dos aparelhos ortopédicos funcionais, de acordo com suas indicações, pode minimizar as discrepâncias dentoesqueléticas, melhorando, consequentemente, a estética facial na primeira fase da dentição mista. O diagnóstico, a correta confecção do aparelho e a cooperação do paciente são a tríade indispensável para que o AAEK seja um coadjuvante no tratamento dessa má oclusão.

**RESULTADOS:**

os casos clínicos apresentaram melhora significativa nos aspectos esquelético, dentário e de perfil, evidenciada pelas análises cefalométricas e, no aspecto clínico, pelas fotografias pré- e trans-tratamento e após a interceptação.

## INTRODUCTION

Class II malocclusion is often associated with one of the following: mandibular retrognathism, anterior displacement of the maxilla, increased vertical dimension of posterior maxilla, mandibular fossa in posterior position, maxillary constriction and a combination of factors. In general, maxilla and mandibular incisors are well-positioned, differently from maxillary incisors which tend to be protrusive.^1^^-^ 4 In Class II skeletal malocclusion, mandibular retrognathism seems to be the major contributing factor.^3^

Kingsley (1879) was the first to use forward positioning of the mandible in orthodontic treatment. The removable appliance developed by the author comprises a continuous labial wire, a bite plane extending posteriorly and molar clasps, and is considered the prototype of functional orthopedic appliances. As he described it, the objective was not to protrude mandibular teeth, but to change or jump the bite in case of an excessively retrusive mandible.^5^

Functional orthopedic appliances have been widely used in Europe since the 1930s,^6^^,^ 7 particularly focusing on changing the muscle conditions that affect mandibular position and function. These appliances, whether fixed or removable, are used to correct Class II malocclusion while improving shape and function of the maxilla and mandible, stimulating natural growth by transduction of forces from muscles to basal bones and dentoalveolar process, affecting the neuromuscular complex, and treating mandibular deficiency.^6^^,^ 8 ^-^ 11 Since forward mandibular growth is often limited by a narrow maxillary arch, functional orthopedics considers correcting sagittal discrepancy by maxillary expansion which allows the mandible to be placed forward.^12^^,^ 13

In mixed dentition, children or preadolescents might develop esthetically unfavorable malocclusion and, for this reason, be exposed intentionally and repeatedly to acts of physical or psychological violence by one person or a group of people (bullying). This might cause victims to feel pain, anxiety and low self-esteem, which significantly affects their psychosocial development.^14 ^The use of functional orthopedic appliances, whenever properly recommended, is able to minimize dentoskeletal discrepancies with consequent improvement in patient's facial esthetics.

Class II, Division 1 malocclusion treatment comprehends one or two phases. In 2-phased treatment, the first phase is carried out in mixed dentition with potential application of maxillary functional orthopedics (MFO), followed by a corrective phase in the early permanent dentition.^15^

This special article aims at reporting five cases of interceptive orthodontics performed with the aid of Klammt's elastic open activator (KEOA) during the first phase of treatment. Clinical outcomes minimized dental and skeletal discrepancies and proved a feasible alternative that contributes to orthodontically treat Class II skeletal malocclusion and Angle Class II, Division 1 malocclusion.

## RECOMMENDATION AND ADVANTAGES

MFO success relies on compliant patients not referred for treatment with tooth extraction, who are short-faced (brachycephalic), with increased posterior facial height, mild to moderate overjet, excess overbite, active facial growth and counterclockwise rotation of the mandible.^6^^,^ 8 ^,^ 10 ^,^ 16 The advantages provided by the activator include: (1) potential for treatment in primary dentition, early or late mixed dentition; (2) appointments spread out to two months or more; (3) tissues are not easily injured; (4) the appliance is used at night which renders it esthetically acceptable and favors hygiene control; and (5) it contributes to eliminate mouth breathing and tongue thrusting habits.^5^

## SIDE EFFECTS AND DISADVANTAGES

Side effects commonly found at treatment completion include posterior open bite,^17^ increased anterior facial height, protrusion of mandibular incisors and proclined maxillary incisors.^1^^,^ 18 ^-^ 21 The disadvantages include: (1) treatment success relies on patient's compliance; (2) activators are of little value in cases of marked crowding; (3) the appliance does not provoke response from older patients; (4) forces exerted on teeth cannot be controlled precisely as in fixed appliances;^5 ^and (5) there is a risk of patients accidently swallowing the appliance.^22^

## KLAMMT APPLIANCE

The appliance developed by Klammt (1969) derived from Andresen and Häupl's appliance, and was termed "open activator" of three different types: the first had an expansion screw with palatal support, used when there was a need for maxillary expansion greater than 3 mm; the second had a one-piece lower appliance combined with a transpalatal arch, used when there was no need for significant expansion; and the third, termed elastic open activator, provided plenty of space for the tongue and could also be used during the day without bringing discomfort to patient's cheeks, lips and tongue. As such, the appliance remains in function without causing any tension and while following all movements performed by the mandible.^23^

## CONSTRUCTION BITE

Appliance manufacture requires a construction bite or working casts mounted in semi-adjustable articulators. A U-shaped construction bite wax is prepared to be inserted between dental arches and acquire the shape of the arch. It should be of adequate width and between 2-3 mm thick. The wax is slightly softened and placed onto the mandibular arch; dentally-guided forward (sagittal) mandibular movement is then performed so as to achieve maximal intercuspation (case 3). During construction bite, forward movement of the mandible does not exceed 10 mm at each stage. Advancement greater than 10 mm requires a second stage, during which a new appliance is manufactured.^24^ Gradual advancement of the mandible demands adaptation to the appliance within a shorter period of time, which favors patient's comfort. Maximum advancement performed at one single stage provides patients with greater discomfort after appliance placement; however, with no further biological effects. Nevertheless, when variables of overjet, overbite and molar and canine relationship are assessed, both types of advancement result in similar improvement.^17^

## APPLIANCE USE PROTOCOL

At the time of appliance placement, patient and parents are informed about the time of appliance use and appliance hygiene, as well as swallowing and speech issues. The appliance should be worn for as long as possible, except during meals and sports practice involving physical contact. At the following appointments, it is possible to assess whether the appliance is being correctly used or not by monitoring patient's speech, swallowing movements and the marks left on the mucosa by buccal archwires, which is an obvious sign of use. Patient's compliance is key to treatment success.

Activation control might be performed every 15 days or on a monthly basis. Adjustments might be rendered necessary so as to provide patient with comfort. Once KEOA treatment objectives are achieved, patients are advised to wear the appliance as a retainer (at night) during a period equivalent to half the active period.

KEOA placement comprehended an initial adaptation period that ranged from two to four weeks. Soon after that, patients were advised to wear the appliance full-time, except during meals and sports practice. Appointments were scheduled every 15 days, with monthly activations of coffin springs (approximately 0.25 mm activation with the aid of a bird beak plier) during treatment.

## TREATMENT OBJECTIVES

Correcting skeletal and dental discrepancies resulting from Class II, Division 1 malocclusion during growth acceleration, and reducing the need for biomechanics during the corrective phase of orthodontic treatment. All patients reported herein were growing patients; however, at different phases.

## DIAGNOSIS, TREATMENT PROGRESS AND INTERCEPTIVE ORTHODONTICS OUTCOMES

## Case report 1

**Figure:**
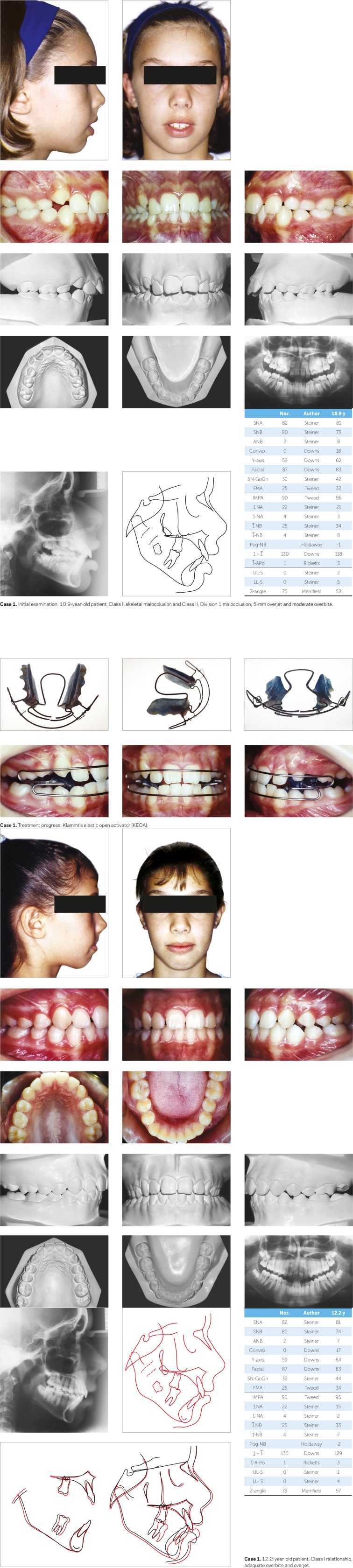
Case 1.

Female 10.9-year-old patient in the second transitional period of mixed dentition. She presented with increased lower facial height and a convex profile, Class II skeletal malocclusion (ANB = 8°) and Class II, Division 1 malocclusion, 6.5-mm overjet and moderate overbite. Cephalometric measurements revealed the patient had a well-positioned maxilla (SNA = 81°), mandibular retrognathism relative to the cranial base (SNB = 73°) and predominantly vertical facial growth pattern (SN.GoGn = 42°). Maxillary incisors were slightly proclined (1-NA = 21°) and retrusive (1-NA = 3°), whereas mandibular incisors were labially proclined (1-NB = 34°) and protrusive (1-NB = 8°). In addition, she presented with lip incompetence and predominantly mouth breathing. 

Skeletal changes resulting from KEOA treatment included mild protrusion of the mandible expressed in a SNB value of 74°, with consequent reduction in the relationship between the maxilla and mandible (ANB = 7°) during treatment. As for facial growth pattern (SN-GoGn = 42° and FMA = 32°), there was a slight increase in the vertical plane of both vectors (SN-GoGn = 44° and FMA = 34°). Maxillary incisors ended up proclined and retrusive, whereas mandibular incisors were slightly proclined and retrusive. Patient's profile was less convex (Z-angle = 57°).

## Case report 2

**Figure:**
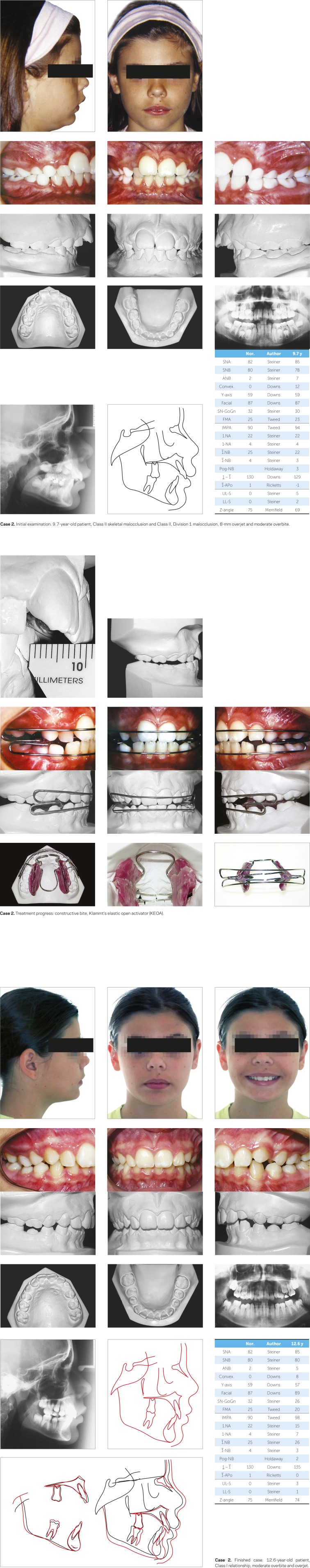
Case 2.

Female 9.7-year-old patient in transitional mixed dentition. She presented with short lower facial height, convex profile, mandibular retrognathism, balanced vertical and horizontal growth patterns (SN.GoGn = 30°; FMA = 23°; Y-axis= 59°), Class II skeletal malocclusion (ANB = 7°) and Class II, Division 1 malocclusion with 9.0-mm overjet and moderate overbite. Maxillary and mandibular incisors were slightly proclined. There was mandibular midline deviation to the right and maxillary constriction in the region of primary molars; however, without posterior crossbite. Palatal inclination of maxillary right lateral incisor. In addition, she presented with lip incompetence and predominantly mouth breathing. 

As for skeletal changes, the maxilla remained in unchanged position (SNA = 85°), since SNA angle remained stable. However, there was an increase in SNB angle (SNB = 80°), which revealed that the mandible was positioned forward, with consequent reduction in the relationship between the maxilla and mandible (ANB = 5°) during treatment. As for facial growth pattern (SN-GoGn = 26° and FMA = 20°), there was a slight decrease in the vertical plane.

Maxillary incisors were proclined and retrusive (1-NA = 15 and 1-NA = 7 mm), whereas mandibular incisors were slightly buccaly proclined (1-NB = 26). There was significant improvement in patient's facial profile, as revealed by Z-angle values (Z = 74°).

## Case report 3

**Figure:**
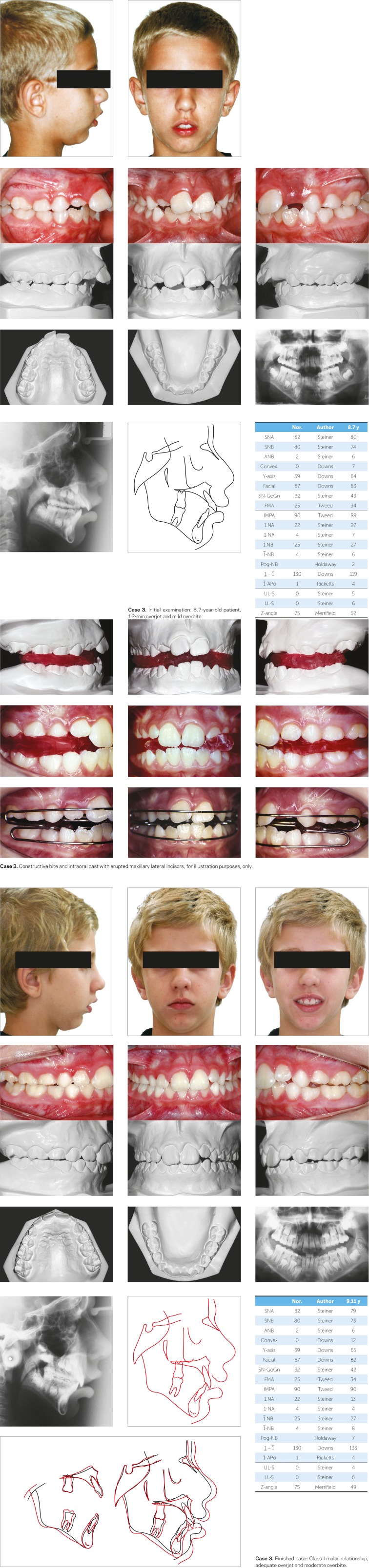
Case 3.

Male 8.7-year-old patient in the first transitional period of mixed dentition. He presented with increased lower facial height and convex profile (Z = 52°), mandibular retrognathism (SNB = 74°) and predominantly vertical growth pattern (Y-axis = 64°, SN-GoGn = 43°). Class II skeletal malocclusion (ANB = 6°), Class II, Division 1 malocclusion, 12-mm overjet and normal overbite. Maxillary (1-NA = 27°) and mandibular incisors (1-NB = 27°) were slightly protrusive. In addition, he presented with predominantly mouth breathing and maxillary constriction; however, without posterior crossbite. Torsiversion of maxillary and mandibular central incisors.

Skeletal changes resulting from KEOA treatment were practically nonexistent, as SNA slightly decreased, which revealed restriction of maxillary anterior displacement with a slight decrease in SNB. This case experienced more marked dental changes in the maxilla, with proclined, retrusive maxillary incisors and mandibular incisors remaining stable.

## Case report 4

**Figure:**
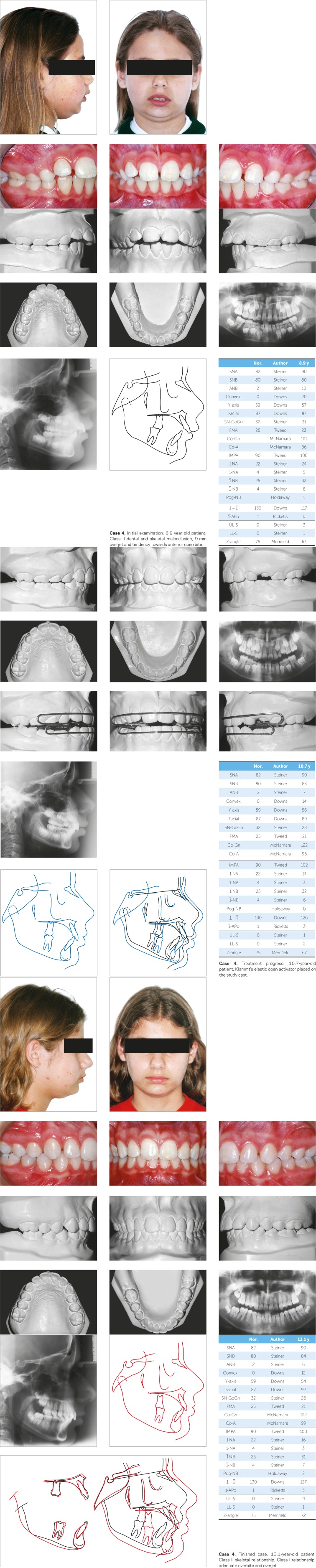
Case 4.

Female 9.9-year-old patient in transitional mixed dentition. She presented with short lower facial height and tendency towards predominantly sagittal growth pattern (Y-axis = 57°, SN-Gn = 31°), lip incompetence and predominantly mouth breathing. Convex profile (Z = 67°). Class II skeletal malocclusion (ANB = 10°) and Class II, Division 1 malocclusion, 9-mm overjet and overbite with a tendency towards anterior open bite. Maxillary prognathism (SNB = 90°), relatively well-positioned maxillary incisors (1-NA = 24°) and mandibular incisors significantly protrusive (1-NB = 32°). Maxillary constriction in the region of primary molars; however, without posterior crossbite, in addition to diastema between maxillary incisors. Skeletal changes and SNA angle analysis of this case suggest no increase in maxillary protrusion and no partial restriction of anterior maxillary displacement. Meanwhile, SNB angle presented with an increase in mandibular protrusion, with consequent reduction in the relationship between the maxilla and mandible during the orthopedic phase of treatment. In terms of patient's horizontal growth pattern, all variables had values within normality. 

Dental changes derived from treatment included marked lingual inclination and retrusion of maxillary incisors, and buccal inclination and protrusion of mandibular incisors. In addition, there was significant improvement in lower facial midlines. 

## Case report 5

**Figure:**
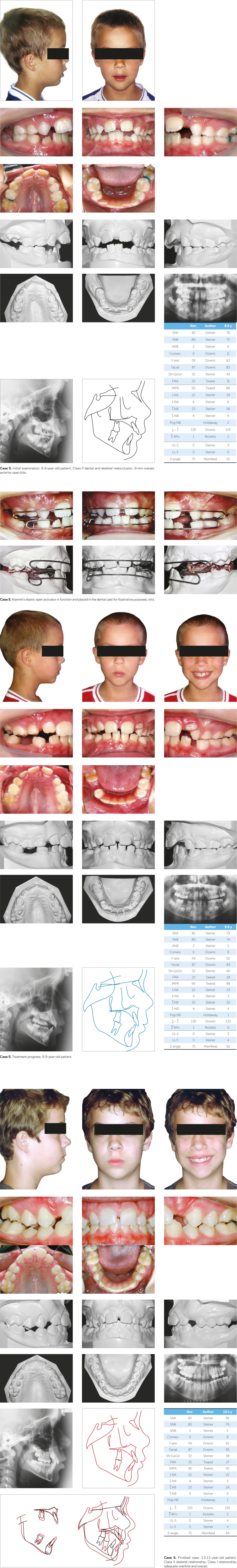
Case 5.

Male 8.9-year-old patient in the first transitional period of mixed dentition. He presented with increased lower facial height, convex profile and mandibular retrognathism (SNB = 72°). Class II skeletal malocclusion (ANBv = 6°), Class II, Division 1 malocclusion, 9-mm overjet and anterior open bite. Protrusive maxillary incisors (1NA = 34°) and proclined mandibular incisors (1NB = 18°). In addition, the patient had a tendency towards vertical growth greater than anteroposterior growth (Y-axis = 63°, SN-GoGn = 43°) and maxillary constriction in the region of primary molars; however, without posterior crossbite. Diastema between maxillary incisors, lack of space for eruption of maxillary lateral incisors and mandibular right canine. Mandibular midline slightly deviated to the right and impaction of teeth #16 and 46 in the distal curvature of primary second molars.

Nine months after treatment onset, Klammt's elastic open activator (KEOA) improved the relationship between the maxilla and mandible, as well as overjet and overbite. In addition, Class I molar relationship was achieved, with space gain that allowed mandibular right second premolar to erupt and considerable change in facial profile.

Post-treatment lateral cephalogram revealed dentoalveolar and skeletal changes, in addition to a decrease in the ANB angle to 5° due to restriction of anterior maxillary growth and mandibular response. It also revealed lingual inclination of maxillary incisors (1-NA = 22°), protrusion of mandibular incisors within normality standards, and improvement in facial profile (Z = 64°). 

The appliance remained in use for another six months, with occasional use during the day going to constant use at night. During the retention phase, permanent teeth erupted and treatment outcomes remained unchanged.

## DISCUSSION

The potential effects produced by correcting Class II, Division 1 malocclusion might derive from one of the following factors: restricted maxillary or dentoalveolar components, increased growth of the mandible or mesial and vertical alveolar growth, anterior relocation of the mandibular fossa, and protrusion of mandibular incisors, thereby correcting overjet.^2^^,^ 6 ^,^ 25 ^,^ 26

The ideal time for malocclusion treatment onset remains controversial. A 2-phased treatment is advocated by some clinicians as advantageous, while others consider it to be a waste of time and money. The 2-phased treatment should be recommended on a case-by-case basis, not as a treatment option to the majority of Class II malocclusion cases. Additionally, it is considered an option only when it provides patients with additional benefits.^15^ All patients reported in the present study gained clinically significant esthetic benefits.

Even though only 0.2% of patients aged between 8 and 11 years old have overjet greater than 10 mm, these children are most likely to be looked down and experience social discrimination. They also present a higher risk of trauma of anterior teeth during accidents due to having protrusive maxillary incisors. Thus, treatment at an early age might have a positive psychological impact over patient's self-esteem. To this end, the resources provided by MFO followed by corrective orthodontics are an option.^27^

MFO is a clinical activity that provides benefits to growing patients, provided that they comply with the use of the appliances (10 to 15 hours a day during 1.5 to 2 years), as illustrated by the cases reported herein. Potential and direction of growth are also important.^28 ^The ideal time for orthopedic appliance use is during the phase of active growth, which allows facial growth pattern to be restores to normality.^6^^,^ 7 ^,^ 8 ^,^ 10 ^,^ 16

In general, as illustrated by the cases reported in the present study, changes produced by KEOA over Class II malocclusion are due to a combination of skeletal and dental factors. There was a reduction in SNA angle, in addition to mandibular protrusion (increased SNB angle), retrusion of maxillary incisors, maintenance of mandibular incisors inclination, unchanged facial vertical dimensions, and improvement in facial profile.

In case 1, the Klammt appliance did not cause any changes in maxillary growth; this basal bone remained stable, with only slight anterior displacement of the mandible. As reported in the literature,^29^ the increase in SN-GoGn and FMA was due to the fact that the appliance was mounted in construction bite with increased interocclusal space between teeth. This process is rather common in functional appliance manufacture.

In case 2, the vertical variables most likely decreased due to counterclockwise mandibular rotation associated with two aspects inherent to the Klammt appliance: impaired eruption of maxillary molars caused by the block of acrylic in the occlusal region; and absence of the same block in the anterior region, which allows greater vertical development of anterior teeth.

In case 3, there was a decrease in the SNA angle, which suggested restriction of anterior maxillary displacement caused by retractor muscles of the mandible, and slight decrease in the SNB angle due to vertical mandibular displacement during facial growth, which caused clockwise rotation of the mandible. These values were already expected due to patient's vertical growth pattern, as indicated by Y-axis, FMA and SN-GoGn variables. This case experienced more marked dental changes in the maxilla, with proclined, retrusive maxillary incisors and mandibular incisors remaining stable.

In case 4, there was restriction of anteroposterior maxillary growth, evinced by a decrease in the SNA angle. According to Webster,^30^ who requotes Blau,^31 ^functional appliances affect the maxilla and mandible at the same time, and are mounted in construction bite, which requires masticatory muscles to act in a different direction (posteriorly), thereby leading to restriction of maxillary growth.

In case 5, the relationship between the maxilla and mandible was effectively restored to normality by the activator, as a result of an increase in mandibular protrusion. Changes were practically nonexistent for the facial growth pattern variables assessed.^32^ Nevertheless, dental changes derived from treatment resulted in proclined and retrusive maxillary incisors, in addition to slight buccal inclination and protrusion of mandibular incisors.

KEOA is particularly effective in contributing to Angle Class II, Division 1 malocclusion treatment. It is recommended to patients with a tendency towards favorable growth, mandibular retrognathism, marked overjet and relatively adequate arch circumference, both lower and upper arches, during the phase of active growth. This is because it results in dentoalveolar changes and improved relationship between the maxilla and mandible, with satisfactory clinical outcomes and minimal correction of skeletal discrepancies restricted to the second phase of treatment performed with a fixed appliance. All the above has been reported for the five cases presented herein.

## FINAL CONSIDERATIONS

Klammt's elastic open activator (KEOA), used to treat Class II, Division 1 malocclusion, achieved the objectives of intercepting or minimizing the existing problem, in addition to reducing the risk of trauma involving maxillary incisors labially proclined and providing patients with psychological benefits and self-esteem. Treatment finishing was performed with fixed orthodontic appliances, which allowed proper function and balance to be achieved, both of which should be part and parcel of treatment planning.
